# Prevalence of Infective Endocarditis among Patients with *Staphylococcus aureus* Bacteraemia and Bone and Joint Infections

**DOI:** 10.3390/microorganisms12020342

**Published:** 2024-02-06

**Authors:** Matthaios Papadimitriou-Olivgeris, Benoit Guery, Pierre Monney, Laurence Senn, Sylvain Steinmetz, Noémie Boillat-Blanco

**Affiliations:** 1 Infectious Diseases Service, Lausanne University Hospital, University of Lausanne, 1011 Lausanne, Switzerland; benoit.guery@chuv.ch (B.G.); laurence.senn@chuv.ch (L.S.); noemie.boillat@chuv.ch (N.B.-B.); 2 Service of Hospital Preventive Medicine, Lausanne University Hospital, University of Lausanne, 1011 Lausanne, Switzerland; 3 Department of Cardiology, Lausanne University Hospital, University of Lausanne, 1011 Lausanne, Switzerland; pierre.monney@chuv.ch; 4 Department of Orthopaedic Surgery and Traumatology, Lausanne University Hospital, University of Lausanne, 1011 Lausanne, Switzerland; sylvain.steinmetz@chuv.ch

**Keywords:** *Staphylococcus aureus*, bloodstream infection, prosthetic joint infection, septic arthritis, vertebral osteomyelitis

## Abstract

We aimed to evaluate the occurrence of infective endocarditis (IE) among patients with bone and joint infections (BJIs) and *Staphylococcus aureus* bacteraemia. This observational study was conducted at Lausanne University Hospital, Switzerland, from 2014 to 2023, and included episodes involving BJI, *S. aureus* bacteraemia, and cardiac imaging studies. The endocarditis team defined IE. Among the 384 included episodes, 289 (75%) involved native BJI (NBJI; 118 septic arthritis, 105 acute vertebral or non-vertebral osteomyelitis, 101 chronic osteitis), and 112 (29%) involved orthopedic implant-associated infection (OIAI; 78 prosthetic joint infection and 35 osteosynthesis/spondylodesis infection). Fifty-one episodes involved two or more types of BJI, with 17 episodes exhibiting both NBJI and OIAI. IE was diagnosed in 102 (27%) episodes. IE prevalence was 31% among patients with NBJI and 13% among patients with OIAI (*p* < 0.001). The study revealed a high prevalence of IE among *S. aureus* bacteraemic patients with NBJI, with notably lower prevalence among those with OIAI.

## 1. Introduction

*Staphylococcus aureus* bacteraemia (SAB) is a common cause of both community and nosocomial acquired bloodstream infections, carrying the potential for rapid progression to severe complications [1,2]. Among these complications, bone and joint infections (BJIs) and infective endocarditis (IE) are the most frequent, with *S. aureus* being the primary causative microorganism for these conditions [3,4,5,6]. Prompt identification of these complications is crucial, as it directly impacts management through targeted antimicrobial treatment and source control interventions when required, ultimately improving patient outcomes [7,8,9].

The likelihood of IE in SAB patients with BJI varies depending on the type of the latter [3,6,10,11]. The link between *S. aureus* IE and native BJIs (NBJIs) is well-established, with IE occurring in up to 33% of bacteraemic NBJI cases [3,10]. Conversely, the correlation between IE and orthopedic implant-associated infection (OIAI) among bacteraemic patients is less explored, showing prevalence rates ranging from 8% to 14% [6,11,12,13]. These associations prompted the inclusion of spondylodiscitis and NBJI in predictive scoring systems used to identify SAB patients at heightened risk of developing IE [4,5]. Despite the observed correlation between BJI and IE, determining which condition is the primary event remains challenging.

The revised Duke clinical criteria proposed by the 2023 European Society Guidelines (ESC) incorporated metastatic osteoarticular infections as a vascular phenomenon [14]. However, despite this inclusion, the guidelines focused exclusively on spondylodiscitis, overlooking other forms of BJI, whether native or implant-associated. This omission underscored the need for further investigation. Therefore, the objectives of this study were to assess the occurrence of IE among *S. aureus* bacteraemic patients presenting with various types of BJIs and compare the differences between NBJIs and OIAIs.

## 2. Materials and Methods

### 2.1. Study Design

This retrospective study was conducted at Lausanne University Hospital, Switzerland, combining two cohorts: (1) an *S. aureus* bacteraemia cohort (retrospective inclusion from January 2015 to December 2021), and (2) a cohort of patients with suspected IE (January 2014 to June 2023; retrospective inclusion from January 2014 to December 2017; prospective inclusion from January 2018 to June 2023). The study was approved by the ethics committee of the Canton of Vaud (CER-VD 2021-02516, CER-VD 2017-02137). Written informed consent was obtained for the prospective cohort, while the ethics committee waived the need for informed consent from the retrospective cohorts.

### 2.2. Patients

Inclusion criteria for the study encompassed adult patients (≥18 years) who had a BJI (NBJI and/or OIAI) AND at least one positive blood culture for *S. aureus*. For the prospective cohort, an additional requirement was the presence of written consent, while for the retrospective cohort, we included all patients who did not have a written refusal for data use. The exclusion criterion was an absence of cardiac imaging studies, such as transthoracic or transesophageal echocardiography (TTE, TEE), ^18^F-Fluorodeoxyglucose Positron Emission Tomography/Computed Tomography (^18^F-FDG PET/CT), or cardiac CT.

Data regarding demographics (age, sex), comorbidities, Charlson Comorbidity Index [15], BJI data (type, contiguous infection, and timing of local symptom onset), systemic symptoms (timing of onset), fever, presence of sepsis or septic shock, cardiac imaging studies, cardiac predisposing factors, embolic events, immunologic phenomena, and laboratory values (white blood cells and C-reactive protein) were retrieved from patients’ electronic health records by an infectious diseases specialist and stored and managed using REDCap (Research Electronic Data Capture; v14.1.2). REDCap is hosted at Lausanne University Hospital. REDCap is a secure, web-based software platform designed to support data capture for research studies [16,17].

### 2.3. Management of SAB

According to internal guidelines, an infectious diseases consultation was performed on a mandatory basis within the same day of blood culture positivity for *S. aureus*. Follow-up blood cultures at 48 h intervals were recommended until negativity. Our internal policy recommended TTE in all cases with exception of nosocomial catheter-related SAB in the absence of risk factors for IE (prior IE, presence of cardiac implantable electronic device or prosthetic valve, persistent bacteraemia for 48 h, or embolic event). TEE was proposed in all patients with community-acquired bacteraemia and in those with nosocomial bacteraemia in the presence of aforementioned risk factors. The endocarditis team determined the necessity for additional cardiac imaging studies, such as ^18^F-FDG PET/CT or cardiac CT, on a case-by-case basis. Among patients with BJIs, blood cultures were recommended even in the absence of systemic symptoms.

### 2.4. Definitions

The diagnosis of different types of BJI was based on published recommendations [18,19,20,21]. OIAI was classified as a contiguous infection if it manifested within three months following orthopedic implant insertion or revision (termed primary postsurgical OIAI). NBJI was considered contiguous if it appeared within three months following joint or bone manipulation. This three-month timeframe aligns with the definition of early-onset PJI, attributed to possible contamination during the operation [22]. Acute BJIs were considered all types of infections, with the exception of chronic osteitis. The timing of local symptom onset in relation to systemic symptom onset was assessed in patients with acute BJI. In episodes where systemic symptoms were not observed, the day of the first positive blood culture was regarded as the onset of systemic symptoms. Episodes were categorized into three groups: (A) episodes where local symptoms began at least four days before systemic symptoms, (B) episodes where local symptoms started between three days before and one day after the initiation of systemic symptoms, and (C) episodes where local symptoms began two days after the onset of systemic symptoms.

The primary outcome was the presence of an IE diagnosis as established by the institution’s endocarditis team at day 60, based on an evaluation of clinical, laboratory, microbiological, imaging, surgical, and histopathological results. Cardiac predisposing factors, embolic events, and immunological phenomena were outlined in the 2015 ESC modified Duke criteria [23]. The definitions of sepsis and septic shock were based on the proposal by the Sepsis-3 International Consensus [24]. Bacteraemia was classified as nosocomial if the first positive blood cultures were obtained more than 48 h after hospital admission. 

### 2.5. Statistical Analysis

Data analysis was performed with SPSS 26.0 (SPSS, Chicago, IL, USA). Categorical variables were analyzed using a chi-square or Fisher exact test, and continuous variables using a Mann–Whitney U test. All statistic tests were two-tailed, and *p* < 0.05 was considered statistically significant. 

## 3. Results

In both cohorts, we identified 394 unique episodes involving SAB and BJI (NBJI and/or OIAI); 10 were excluded due to a lack of cardiac imaging. Among the 384 included episodes, 289 (75%) involved NBJI, including 118 cases of septic arthritis, 105 cases of acute vertebral or non-vertebral osteomyelitis, and 101 cases of chronic osteitis; 35 presented two different types of NBJI. OIAI was diagnosed in 112 (29%) episodes, comprising 78 cases of prosthetic joint infection (PJI) and 35 cases of osteosynthesis/spondylodesis infection; one episode presented with both PJI and osteosynthesis/spondylodesis infection. Seventeen episodes exhibited both NBJI and OIAI. Table 1 depicts the characteristics of the 384 episodes with SAB and BJIs, categorized into episodes with NBJI, OIAI, and both NBJI and OIAI. In total, 369 different localizations of acute BJIs (BJIs excluding chronic osteitis) were observed; 139 septic arthritis, 107 acute vertebral or non-vertebral osteomyelitis, 88 PJI, and 36 osteosynthesis/spondylodesis infections (Table 2).

Methicillin resistance was observed in 23 (6%) episodes. No difference was observed between episodes with NBJI and OIAI (7% vs. 5%; *p* 0.807). Additionally, resistance to clindamycin, quinolones, doxycycline, rifampicin, and cotrimoxazole was noted in 51 (13%), 25 (7%), eight (2%), eight (2%), and six (1%) episodes, respectively.

By excluding 17 episodes with both NBJI and OIAI, episodes involving NBJI more commonly exhibited cardiac predisposing factors compared to OIAI (18% vs. 8%; *p* 0.032). This distinction was primarily attributed to a higher incidence of intravenous (IV) drug use among NBJI episodes (10% vs. 1%; *p* 0.002). Additionally, patients with NBJI were found to suffer with prolonged bacteraemia lasting at least 48 h more frequently (40% vs. 24%; *p* 0.006). No significant difference was observed between NBJI and OIAI patients concerning the presence of systemic symptoms or fever. 

TTE was conducted in 354 (92%), TEE in 234 (61%), ^18^F-FDG PET/CT in 49 (13%), and cardiac CT in 4 (1%) episodes. IE was diagnosed in 102 (27%) episodes. Among these, 79 (77%) were native valve IE, 13 (13%) were prosthetic valve IE, and 20 (20%) were related to CIED-lead infections. In 132 (43%) episodes the aortic valve was affected, in 122 (40%) the mitral, in 42 (14%) the tricuspid, and in 4 (1%) the pulmonary; in 27 patients (9%) multiple valves were affected. Vegetation was observed in 191 (63%) episodes, and among these episodes, the vegetation was larger than 10 mm in 94 (49%). In 44 (14%) episodes an intracardiac abscess was observed, in 31 (10%) valve perforation or dehiscence of prosthetic valve, and in 5 (2%) intracardiac aneurysm, pseudoaneurysm, or fistula. In 36 (12%) episodes a hypermetabolic intracardiac lesion was observed via ^18^F-FDG PET/CT. Table 3 depicts the characteristics associated with infective endocarditis among 384 episodes with SAB and BJI.

IE prevalence was 31% (91/289) among patients with NBJI and 13% (14/112) among patients with OIAI (*p* < 0.001). Among patients with NBJI, IE was diagnosed in 40% with septic arthritis, 37% with acute vertebral or non-vertebral osteomyelitis, and 18% with chronic osteitis (Table 2). Among patients with OIAI, IE was diagnosed in 23% with osteosynthesis/spondylodesis infection, and 9% with PJI. In patients with PJI infection as the sole OA infection, the prevalence of IE was 5% (3/63). Episodes involving multiple localizations of BJI were more prevalent in the IE group (26% vs. 13%; *p* 0.004). Conversely, episodes with contiguous BJI were less frequently associated with IE (17% vs. 34%; *p* 0.007).

Concerning the different prediction scores for the identification of patients with *S. aureus* bacteraemia at high-risk for IE, VIRSTA and LAUSTAPHEN (LAUsanne *STAPHylococcus aureus* ENdocarditis) correctly categorized 97% of IE patients in the high-risk group. LAUSTAPHEN fared better than VIRSTA, as it categorized in the high-risk group 58% of episodes without IE, compared to 83% by VIRSTA. The PREDICT score classified 94% of IE episodes as high-risk; however, it also classified 90% of episodes without IE as high-risk. The POSITIVE score failed to classify 24% of IE episodes as high-risk (Table 3).

**Figure 1:**
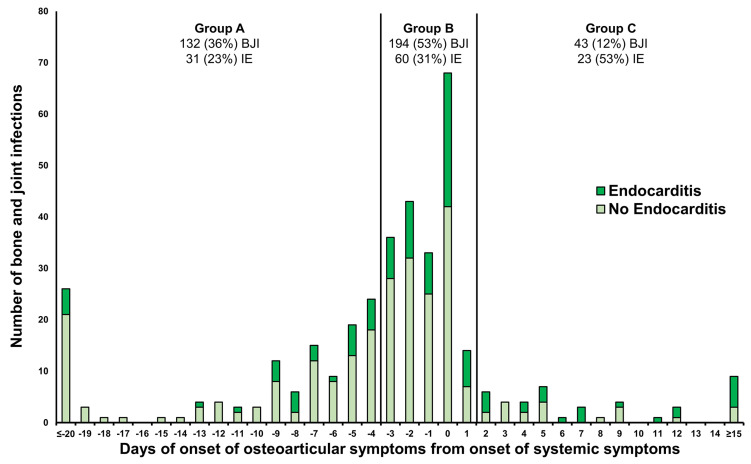
The risk of infective endocarditis in 369 acute bone joint infections based on the delay of local symptoms onset relative to the onset of systemic symptoms. Group A: local symptoms onset at least 4 days before systemic symptoms; Group B: local symptoms onset between 3 days before and 1 day after the initiation of systemic symptoms; Group C: local symptoms onset 2 days after the onset of systemic symptoms. BJI: bone and joint infection; IE: infective endocarditis.

Concerning the timing of local (osteoarticular) symptoms onset relative to the initiation of systemic symptoms among 369 different localizations of acute BJIs (Figure 1), the distribution was as follows: 132 (36%) BJIs fell into Group A (local symptoms began at least four days before systemic symptoms), 194 (53%) BJIs into Group B (local symptoms starting between three days before and one day after systemic symptoms), and 43 (12%) BJIs into Group C (local symptoms beginning two days after systemic symptoms). The proportion of infective endocarditis (IE) cases differed significantly among the three groups (*p* 0.001), constituting 23% (31 BJIs) in Group A, 31% (60 BJIs) in Group B, and 53% (23 BJIs) in Group C, respectively.

## 4. Discussion

Although the link between IE and BJI is acknowledged, certain questions remain unresolved [10,25,26,27,28,29,30,31,32]. Most studies have focused primarily on NBJI, particularly in cases of vertebral osteomyelitis [25,26,27,28,31]. Additionally, there is a scarcity of data concerning the impact of multiple BJIs [10], contiguous BJI [10], and the timing of local symptoms onset on the risk of developing IE [33].

In the present study, we confirmed a relatively high proportion (27%) of patients with SAB and BJI also had IE, consistent with findings from previous studies (11–33%) [3,10]. This association was notably more pronounced among patients with septic arthritis, where 40% were diagnosed with IE, and among those with acute vertebral or non-vertebral osteomyelitis, where 37% were found to have IE. While spondylodiscitis was recognized by the 2023 ESC guidelines as being associated with IE, to an extent that it was included in the minor Duke vascular phenomena criterion, septic arthritis, which bears a similar risk of IE, was overlooked [4,14].

The significance of vertebral osteomyelitis was also underscored by its inclusion as a parameter in the VIRSTA score, which aids in assessing the risk of IE among SAB patients [4]. In the LAUSTAPHEN score, serving a similar purpose and exhibiting comparable diagnostic performance to VIRSTA, NBJI, including septic arthritis, acute vertebral and non-vertebral osteomyelitis, was incorporated as a parameter [4,5]. In this study, VIRSTA and LAUSTAPHEN had the highest rate (97%) of categorizing IE patients in the high-risk group, compared to PREDICT (94%) and POSITIVE (76%). This makes the former two more suitable for clinical practice, with LAUSTAPHEN being superior in terms of misclassifying patients without IE in the high-risk group. This superiority translates to a reduced number of echocardiograms needed to achieve the same result. Nonetheless, no score can substitute clinical judgment; instead, they should be used in conjunction with it.

Another significant finding is the bidirectional complication potential between IE and BJI, an aspect not thoroughly explored in prior research. In this study, among acute BJI episodes, 42% exhibited local symptoms preceding the onset of systemic symptoms by at least four days, with the percentage being lower (33%) among IE episodes compared to non-IE episodes (33% vs. 46%; *p* 0.041). This challenges the inclusion of metastatic osteoarticular infections in the vascular phenomena criterion by the 2023 ESC guidelines, alongside other embolic events [14,34]. This is because, in one-third of patients, BJI occurred prior to the onset of IE symptoms. Only 10% of episodes displayed local symptoms emerging at least two days after the onset of systemic symptoms (19% in IE episodes vs. 6% in non-IE; *p* 0.001), highlighting that BJI could potentially act as an embolic event in a small subset of patients.

In the present study, an association between IE and patients presenting multiple localizations of BJI was observed. This correlation was previously demonstrated in a study involving bacteraemic patients with septic arthritis, where a higher prevalence of multifocal septic arthritis was found among patients with positive echocardiography compared to those with negative results [10]. Conversely, contiguous BJIs showed a lower frequency of correlation with IE, consistent with prior findings among patients with bacteraemic septic arthritis [10].

We observed a lower rate of IE in patients with OIAI (13%) compared to those with NBJI (31%). This difference was even more pronounced in patients with isolated PJI (5%). In previous studies, the proportion of patients with SAB and PJI that developed IE was variable (11–18%) [6,11]. The discrepancy in these rates might be explained by a low prevalence of cardiac predisposing factors, such as cardiovascular implantable electronic devices, among patients with PJI in our study. Specifically, only 4% of PJI episodes involved a cardiovascular implantable electronic device in our study, compared to 23% in Rakow et al. [6]. The differing prevalence of IE between patients with NBJI and OIAI might be explained by the higher percentage of IV drug use in the former group and a higher bacterial load in patients with NBJI, as shown by the longer duration of bacteraemia and the higher rate of two or more positive blood culture sets in NBJI compared to PJI patients [35,36]. Another potential explanation for the variation in IE prevalence between patients with NBJI and OIAI could be attributed to the nature of the pathogen itself. Small colony variants, typically linked with OIAI and particularly PJI, are less frequently associated with IE [37].

While the study has several limitations, particularly being a single-center retrospective study, it stands out due to the higher number of included patients compared to previous research [3,30,38]. Importantly, unlike earlier studies where only a small percentage of patients underwent cardiac imaging studies [3,6,10,11], all patients in this study underwent such investigations. Another potential limitation arises from the lack of blood culture performance in BJI patients. However, this bias is partly mitigated by our institution’s practice of recommending blood cultures for acute BJI patients, even in the absence of systemic symptoms. Additionally, patients with OIAI are admitted to the septic surgery service, where infectious diseases specialists are integral, ensuring blood cultures are often conducted before initiating antibiotic treatment.

## 5. Conclusions

In conclusion, the study yielded two important findings. Firstly, IE was found in a considerable proportion of patients with SAB and NBJI, especially in cases of septic arthritis and acute vertebral and non-vertebral osteomyelitis. This underscores the importance of echocardiographic screening of bacteraemic patients with NBJI. To aid clinicians, prediction rules for diagnosing IE, such as VIRSTA or LAUSTAPHEN, could be utilized alongside clinical judgment to identify patients who are at a heightened risk for IE. Secondly, the prevalence of IE was lower in patients with OIAI compared to those with NBJI, particularly in those with isolated PJI. A prospective study is required to evaluate whether a good-quality TTE in patients with SAB and isolated PJI could be sufficient to safely exclude IE.

## Figures and Tables

**Table 1:** Characteristics of 384 episodes with *S. aureus* bacteraemia and bone and joint infection, categorized into episodes with native bone and joint infection, orthopedic implant-associated infection, and both native bone and joint infection and orthopedic implant-associated infection.

	NBJI (n = 272)		OIAI (n = 95)		p *	Both NBJI and OIAI (n = 17)	
Demographics							
Male sex	209	77%	61	64%	0.021	9	53%
Age (years)	70	56–79	70	63–78	0.545	73	69–74
Charlson Comorbidity Index	5	2–7	4	2–6	0.021	5	4–7
Setting of infection onset							
Non nosocomial (community or healthcare-associated)	237	84%	84	88%	0.323	15	88%
Cardiac predisposing factors	48	18%	8	8%	0.032	2	12%
IV drug use	28	10%	1	1%	0.002	0	0%
Prosthetic valve IE	15	6%	4	4%	0.791	2	12%
Cardiovascular Implantable Electronic Device	34	13%	5	5%	0.053	1	6%
Microbiological data							
Two or more positive blood culture sets	224	82%	69	73%	0.053	15	88%
Methicillin-resistance	18	7%	5	5%	0.807	0	0%
Prolonged bacteraemia (≥48 h)	108	40%	23	24%	0.006	12	71%
Clinical presentation							
Systemic symptoms	248	91%	87	92%	1.000	13	77%
Fever (temperature >38 °C)	215	79%	81	85%	0.228	13	77%
Sepsis	121	45%	39	41%	0.631	7	41%
Septic shock	36	13%	11	12%	0.859	3	18%
NBJI	272	100%	0	0%	-	17	100%
Acute NBJI	190	70%	-	-	-	12	71%
Septic arthritis	111	41%	-	-	-	7	41%
Acute osteomyelitis (vertebral and not vertebral)	98	36%	-	-	-	7	41%
Chronic osteitis	96	35%	-	-	-	5	29%
OIAI	0	0%	95	100%	-	17	100%
Prosthetic joint infection	-	-	64	67%	-	14	82%
Osteosynthesis or spondylodesis infection	-	-	32	34%	-	3	18%
Contiguous BJI (n = 295)	48	26%	35	37%	0.073	2	12%
Timing of local symptoms among acute BJI (n = 302)							
At least 4 days before systemic symptoms onset	80	42%	40	42%	1.000	6	35%
At least 2 days after systemic symptoms onset	25	13%	1	1%	<0.001	3	18%
Laboratory data							
White blood cells (×10^9^/L) (n = 370)	13	10–17	13	10–18	0.773	15	11–18
C-reactive protein (mg/L) (n = 195)	235	149–320	270	170–334	0.075	333	167–387
Cardiac imaging studies							
TTE	254	93%	86	91%	0.366	14	82%
TEE	164	60%	53	56%	0.468	17	100%
^18^F-FDG PET/CT or cardiac-CT	42	15%	6	6%	0.022	1	6%
Immunologic phenomena	4	2%	0	0%	0.576	1	6%
Embolic events	50	18%	4	4%	<0.001	2	12%
Infective endocarditis	88	32%	11	12%	<0.001	3	18%

Data are depicted as number and percentage or median and Q1–3. ^18^F-FDG PET/CT: 18-fluorodeoxyglucose positron emission tomography computed tomography; BJI: bone and joint infection; NBJI: native bone and joint infection; TTE: transthoracic echocardiography; TEE: transesophageal echocardiography; OIAI: orthopedic implant-associated infection. * Comparison between episodes that had NBJI or OIAI, excluding the 17 episodes with both NBJI and OIAI.

**Table 2:** Characteristics of patients with *S. aureus* bacteraemia and different types of bone and joint infections categorized as septic arthritis, acute osteomyelitis (vertebral or non-vertebral), chronic osteitis, prosthetic joint infection, and osteosynthesis or spondylodesis infection.

	Septic Arthritis (n = 118)		Acute Osteomyelitis (Vertebral and Non-Vertebral) (n = 105)		Chronic Osteitis (n = 101)		Prosthetic Joint Infection (n = 78)		Osteosynthesis/Spondylodesis Infection (n = 35)	
Demographics										
Male sex	86	73%	73	70%	83	82%	51	65%	20	57%
Age (years)	69	51–79	67	55–75	72	64–78	71	66–79	68	61–75
Charlson Comorbidity Index	4	2–6	4	2–6	7	5–8	4	3–6	4	2–6
Setting of infection onset										
Non nosocomial (community or healthcare-associated)	103	87%	87	83%	82	81%	73	94%	27	77%
Cardiac predisposing factors	19	16%	22	21%	14	14%	4	5%	6	17%
IV drug use	13	11%	17	16%	2	2%	0	0%	1	3%
Prosthetic valve IE	6	5%	5	5%	7	7%	3	4%	3	9%
Cardiovascular Implantable Electronic Device	11	9%	7	6%	19	19%	5	6%	1	3%
Microbiological data										
Two or more blood cultures positive	99	84%	90	86%	80	79%	56	72%	29	83%
Methicillin-resistance	2	2%	10	10%	8	8%	2	3%	3	9%
Prolonged bacteraemia (≥48 h)	58	49%	63	60%	21	21%	27	35%	9	26%
Clinical presentation										
Systemic symptoms	106	90%	95	91%	95	94%	68	87%	34	97%
Fever (temperature >38 °C)	85	72%	86	82%	86	85%	65	83%	18	51%
Sepsis	58	49%	48	46%	42	42%	29	37%	18	51%
Septic shock	23	20%	15	14%	10	10%	10	13%	4	11%
NBJI	118	100%	105	100%	101	100%	14	18%	3	9%
Acute NBJI	118	100%	105	100%	14	14%	11	14%	1	3%
Septic arthritis	118	100%	21	20%	6	6%	7	9%	0	0%
Acute osteomyelitis (vertebral and not vertebral)	21	18%	105	100%	8	8%	6	8%	1	3%
Chronic osteitis	6	5%	8	8%	101	100%	3	4%	2	6%
OIAI	7	6%	7	7%	5	5%	78	100%	35	100%
Prosthetic joint infection	7	6%	6	6%	3	3%	78	100%	1	3%
Osteosynthesis or spondylodesis infection	0	0%	1	1%	2	2%	1	1%	35	100%
Contiguous BJI (n = 295)	24	21%	25	24%	-	-	14	18%	23	66%
Timing of local symptoms among acute BJI (n = 302)										
At least 4 days before systemic symptoms onset	48	41%	47	45%	-	-	37	47%	10	29%
At least 2 days after systemic symptoms onset	24	0%	12	11%	-	-	5	5%	0	0%
Laboratory data										
White blood cells (×10^9^/L) (n = 328)	13	10–16	13	9–18	13	10–18	14	10–18	12	11–18
C-reactive protein (mg/L) (n = 195)	264	168–332	280	137–336	204	142–314	288	194–338	231	154–351
Cardiac imaging studies										
TTE performed	109	92%	96	91%	93	92%	72	92%	29	83%
TEE performed	87	74%	73	70%	50	50%	54	69%	17	49%
^18^F-FDG PET/CT or cardiac-CT performed	14	12%	23	22%	11	11%	5	6%	3	9%
Immunologic phenomena	5	4%	2	2%	0	0%	1	1%	0	0%
Embolic events	26	22%	23	22%	11	11%	4	5%	2	6%
Infective endocarditis	47	40%	39	37%	18	18%	7	9%	8	23%

Data are depicted as number and percentage or median and Q1–3. ^18^F-FDG PET/CT: 18-fluorodeoxyglucose positron emission tomography computed tomography; BJI: bone and joint infection; NBJI: native bone and joint infection; TTE: transthoracic echocardiography; TEE: transesophageal echocardiography; OIAI: orthopedic implant-associated infection.

**Table 3:** Characteristics associated with infective endocarditis among 384 episodes with *S. aureus* bacteraemia and bone and joint infection.

	No IE (n = 282)		IE (n = 102)		*p*
Demographics					
Male sex	201	71%	78	77%	0.365
Age (years)	70	60–78	70	59–78	0.901
Charlson Comorbidity Index	4	2–7	5	3–8	0.393
Setting of infection onset					
Non nosocomial (community or healthcare-associated)	237	84%	99	97%	0.344
Cardiac predisposing factors	27	10%	31	30%	<0.001
IV drug use	17	6%	12	12%	0.079
Prosthetic valve IE	6	2%	15	15%	<0.001
Cardiovascular Implantable Electronic Device	16	6%	24	24%	<0.001
Microbiological data					
Two or more positive blood culture sets	209	74%	99	97%	<0.001
Methicillin-resistance	17	6%	6	6%	1.000
Prolonged bacteraemia (≥48 h)	76	27%	67	65%	<0.001
Clinical presentation					
Systemic symptoms	254	90%	95	93%	0.426
Fever (temperature >38 °C)	227	81%	82	80%	1.000
Sepsis	107	38%	60	59%	<0001
Septic shock	20	7%	30	29%	<0.001
NBJI	198	70%	91	89%	<0.001
Acute NBJI	125	44%	77	75%	<0.001
Septic arthritis	71	25%	47	46%	<0.001
Acute osteomyelitis (vertebral and not vertebral)	66	23%	39	38%	0.006
Chronic osteitis	83	29%	18	18%	0.025
OIAI	98	35%	14	14%	<0.001
Prosthetic joint infection	71	25%	7	7%	<0.001
Osteosynthesis or spondylodesis infection	27	10%	8	8%	0.692
Contiguous BJI (n = 295)	70	34%	15	17%	0.007
Multiple BJI localizations	36	13%	26	26%	0.004
Timing of local symptoms among acute BJI (n = 302)					
At least 4 days before systemic symptoms onset	97	46%	29	33%	0.041
At least 2 days after systemic symptoms onset	12	6%	17	19%	0.001
Laboratory data					
White blood cells (×10^9^/L) (n = 370)	13	10–18	12	10–17	0.413
C-reactive protein (mg/L) (n = 195)	233	151–322	272	184–340	0.035
Immunologic phenomena	2	0.7%	3	3%	0.119
Embolic events	6	2%	50	49%	<0.001

Data are depicted as number and percentage or median and Q1–3. ^18^F-FDG PET/CT: 18-fluorodeoxyglucose positron emission tomography computed tomography; BJI: bone and joint infection; NBJI: native bone and joint infection; TTE: transthoracic echocardiography; TEE: transesophageal echocardiography; OIAI: orthopedic implant-associated infection.

## Data Availability

The data that support the findings of this study are available from the corresponding author upon reasonable request.
